# Inside out: Taking inpatient care home

**DOI:** 10.1192/j.eurpsy.2021.1930

**Published:** 2021-08-13

**Authors:** C. Cunha, R. Valido, E. Machado

**Affiliations:** 1 Psychiatry Department, Magalhães Lemos Hospital, Porto, Portugal; 2 Psychiatry Department, Senhora da Oliveira Hospital, Guimarães, Portugal

**Keywords:** home hospitalization, community-focused care model, inpatient care

## Abstract

**Introduction:**

Home hospitalization is an alternative to conventional hospitalization in several areas of medicine. In Portugal, we are now starting to think about its implementation in Psychiatry, given the positive experience of its use in other countries.

**Objectives:**

Understand the advantages and disadvantages of a home hospitalization model and its logistical and clinical framework in an integrated community-focused care model.

**Methods:**

We performed a literature review using Pubmed databases and UpToDate on home hospitalization, inpatient care and community-focused care model

**Results:**

We have found reports of centers with experience in home hospitalization in Psychiatry, but there is still a notable lack of studies in this area. There is a discrepancy between the care needs of patients and the existence of community services for the treatment of mental illness. Home hospitalization is considered when there is partial remission of the symptomatology that motivated the hospitalization. Albeit demanding inclusion criteria limit eligible patients, there are several advantages with this hospitalization model: 1) it favors agility in the transition from hospital to home, with direct observation of contextual factors that may influence psychiatric decompensation, 2) integrates the patient in his natural environment, promoting his autonomy,; 3) allows psychoeducation of the family; 3) guarantees the continuity of the therapeutic process initiated in the hospital, 4) optimizes resources and cost-effectiveness, 5) prevents relapses and the “revolving-door “phenomenon.

**Conclusions:**

We have found that a model of home hospitalization is a valuable element that should be included in an integrated system of psychiatric care.

**Disclosure:**

No significant relationships.

